# Beyond nighttime symptoms: acupuncture for daytime dysfunction improvement in insomnia—a meta-analysis

**DOI:** 10.3389/fneur.2026.1752313

**Published:** 2026-03-03

**Authors:** Peiqi Li, Qianwen Yang, Jian Pei, Charles Savona Ventura, Linda Zhong, Jie Ma, Qinhui Fu

**Affiliations:** 1 Longhua Hospital, Shanghai University of Traditional Chinese Medicine, Shanghai, China; 2 Department of Obstetrics & Gynaecology, University of Malta, Msida, Malta; 3 Biomedical Sciences and Chinese Medicine, School of Biological Sciences, Nanyang Technological University, Singapore, Singapore

**Keywords:** acupuncture, daytime function, electroacupuncture, insomnia, meta-analysis

## Abstract

Insomnia features include persistent difficulties in sleep initiation and sleep maintenance, leading to significant daytime dysfunction and diminished quality of life. While acupuncture is increasingly utilized in the management of insomnia, its specific efficacy in alleviating daytime dysfunction remains inadequately substantiated. This systematic review aims to address this evidence gap. A total of 5,037 articles from 6 electronic databases were searched and screened. Data analysis was performed using Review Manager 5.4 software and Stata 13. The Cochrane tool for assessing the risk of bias in randomized trials (RoB2) and GRADE were used to evaluate the quality of the RCTs and the evidence. Eighteen randomized controlled trials (RCTs) encompassing a total of 1,767 patients were analyzed. Data of the Montreal Cognitive Assessment (MoCA), Auditory Verbal Memory Test (AVMT), and Fatigue Scale-14 (FS-14) scores were extracted and aggregated, along with the scores of the Pittsburgh Sleep Quality Index (PSQI) and Insomnia Severity Index (ISI). Moderate-quality evidence showed that acupuncture therapies could not only significantly reduce insomnia severity but also enhance memory ability and relieve cognitive impairment. Low-quality evidence presented that acupuncture could improve sleep quality and reduce fatigue in patients with chronic primary insomnia. Although heterogeneity was observed across several synthesized outcomes, the results were robust.

**Systematic review registration:**

The research protocol was registered in PROSPERO (ID: CRD42023442722).

## 1 Introduction

Insomnia is a prevalent public health problem, affecting over 20% of adults (1). Its features include persistent difficulties in sleep initiation, sleep maintenance, and early awakenings (2). These sleep problems could lead to multiple forms of daytime impairment (3), such as fatigue, depressed mood or irritability, poor memory, and cognitive impairment (4). These symptoms seriously affect patients’ work efficiency and quality of life, which are the main reasons patients seek treatment (5).

Currently, pharmacological therapy remains one of the main methods for managing insomnia (6–8). However, the existing approach may cause substantial risks as Z-drugs pose next-day impairment (9) through the suppression of glymphatic activity (10). In light of this, alternative therapies, which can relieve nighttime and daytime symptoms at the same time, are urgently needed.

Emerging evidence supports acupuncture as a promising alternative intervention. Existing meta-analyses (11–14) have confirmed the beneficial effects of various acupuncture-related therapies on primary and secondary insomnia. Nonetheless, these reviews have mainly focused on nighttime sleep indicators, such as the Pittsburgh Sleep Quality Index (PSQI), without assessing their impact on daytime function.

Therefore, this study aims to evaluate the efficacy of acupuncture on daytime symptoms of patients with insomnia and to provide evidence-based interventions for clinical practice.

## 2 Methods and materials

This review was conducted following the Preferred Reporting Items for Systematic Reviews and Meta-Analyses (PRISMA) guidelines. The research protocol was registered in PROSPERO (ID: CRD42023442722).

### 2.1 Search strategy

Six databases, Embase, PubMed, Web of Science, CNKI, VIP Database, and Wanfang Database, were searched from their inception to 1 September 2025. Articles were limited to English and Chinese. The search strategy used Medical Subject Headings (MeSH) terms related to acupuncture and insomnia. Table 1 summarizes the complete PubMed search strategy. The same terminologies were used in the English and Chinese databases (see Supplementary material 1). Additionally, the Cochrane Central Register of Controlled Trials (CENTRAL), SinoMed, PROSPERO, and INPLASY databases were searched to identify relevant literature. Protocols, reviews, pilot studies, case reports, case series, editorials, surveys, animal experiments, notes, letters, and conference reports were excluded.

**Table 1:** Search strategy (taking the PubMed database for example).

Number	Search items
#1	((((((“Acupuncture”[MeSH]) OR “Acupuncture Points” [MeSH]) OR “Acupuncture Therapy”[MeSH]) OR “Dry Needling”[MeSH]) OR “Trigger Points”[MeSH]) OR “Meridians”[MeSH])
#2	(((((((((((acupuncture[Title/Abstract])) OR (acupuncture points[Title/Abstract])) OR (acupuncture therapy[Title/Abstract])) OR (body acupuncture[Title/Abstract])) OR (coiling dragon needling[Title/Abstract])) OR (dermal needle[Title/Abstract])) OR (dry needling[Title/Abstract])) OR (Electro-acupuncture[Title/Abstract])) OR (point injection[Title/Abstract])) OR (trigger points[Title/Abstract])) OR (meridians[Title/Abstract])
#3	(((((((((((((((chronic insomnia[MeSH Terms]) OR (familial fatal, insomnia[MeSH Terms])) OR (chronic insomnia[MeSH Terms])) OR (primary insomnia[MeSH Terms])) OR (Disorders of Initiating and Maintaining Sleep[MeSH Terms])) OR (insomnia[MeSH Terms])) OR (early awakening[MeSH Terms])) AND (awakening, early[MeSH Terms])) OR (nonorganic insomnia[MeSH Terms])) OR (insomnia, nonorganic[MeSH Terms])) OR (transient insomnia[MeSH Terms])) OR (rebound insomnia[MeSH Terms])) OR (dysfunction, sleep initiation[MeSH Terms])) OR (sleeplessness[MeSH Terms])) OR (insomnia disorder[MeSH Terms])) OR (insomnia, psychophysiological[MeSH Terms])
#4	“insomnia s”[All Fields] OR “sleep initiation and maintenance disorders”[MeSH Terms] OR (“sleep”[All Fields] AND “initiation”[All Fields] AND “maintenance”[All Fields] AND “disorders”[All Fields]) OR “sleep initiation and maintenance disorders”[All Fields] OR “insomnia”[All Fields] OR “insomnias”[All Fields] OR (“sleep wake disorders”[MeSH Terms] OR (“sleep”[All Fields] AND “wake”[All Fields] AND “disorders”[All Fields]) OR “sleep wake disorders”[All Fields] OR (“sleep”[All Fields] AND “disorder”[All Fields]) OR “sleep disorder”[All Fields])
#5	#1 OR #2
#6	#3 OR #4
#7	#5 AND #6

### 2.2 Inclusion and exclusion criteria

Inclusion criteria: (1) The study was a randomized controlled trial (RCT) that exclusively used either manual acupuncture or electroacupuncture as an experimental intervention, and used sham acupuncture, wait list control, or drugs as control intervention, (2) the participants were diagnosed with chronic insomnia according to published guidelines, (3) outcomes measured in the study should include at least either the PSQI or Insomnia Severity Index (ISI) score, and (4) the article was in either English or Chinese.

Exclusion criteria include the following: (1) non-comparative studies, (2) duplicate studies (3) studies focusing on secondary insomnia, and studies in which patients were diagnosed with cancer, anxiety, depression, or other psychological illnesses that may lead to secondary insomnia, (4) studies that compared the effects of different acupoints, or used other acupoints on the meridians without the effect of treating insomnia as a control, (5) studies that did not report the selected acupoints, (6) studies without available data on preoperative and postoperative outcomes or those outcomes that could not be calculated using a specific formula, and (7) studies that did not report the randomization process, and studies that did not report ethical statements or registration numbers.

There were no restrictions on sex, needle material, treatment course, or needle retention time.

### 2.3 Study identification and data extraction

After a systematic search, two authors (Peiqi Li and Qianwen Yang) conducted a preliminary screening of the titles, abstracts, and keywords using the Citation Management Software EndNote X9. Subsequently, potentially eligible full texts were obtained and read for article re-screening, and the data were cross-checked. Any disputes were settled by a third reviewer (Qinhui Fu) or by consensus between the two reviewers. When the studies needed to be excluded, the exclusion reasons were recorded. Subsequently, the two reviewers screened the text and extracted data from the eligible studies independently in duplicate, which are detailed in Table 2. The extracted information is listed as follows: year of publication, author, sample size, sex, interventions, outcomes, acupoints selected, treatment frequency, and adverse effects. The missing data were obtained by the reviewers by inquiring with the authors of the original study via email.

**Table 2:** Basic characteristics of includes articles.

Included studies	Control group			Experimental group				Outcome measurements^a^
	Sample size	Intervention	Frequency of treatment	Sample size	Intervention	Frequency of treatment	Acupoints	
Chung et al. (2018) (17)	32	Waiting	NA^b^	96	Electroacupuncture	Three times a week for 3 weeks	PC6, HT7 and SP6, Sishencong, Anmian, and unilaterally at GV20 and GV29	1,2,3,5,11, Sleep diary and actigraph measures, anxiety, depression, fatigue, sleepiness and functional measures, and MFI scores
Das et al. (2022) (21)	20	Sham acupuncture	Daily 30 min for 2 weeks	20	Manual acupuncture	Daily 30 min for 2 weeks	HT7, GV20, GV29	1,5, subjective sleep quality, daytime dysfunction, sleep duration, sleep latency, sleep disturbance, habitual sleep efficiency
Wu et al. (2023) (25)	45	Medication: Estazolam (1 mg)	Nightly for 4 weeks	45	Manual acupuncture	Once a day, 6 times a week for 4 weeks	HT7, SP6, Sishencong	1,6,10, mini-mental state examination (MMSE)
Feng et al. (2020) (18)	45	Sham acupuncture	5 times a week, for 4 weeks	45	Manual acupuncture	Continued for 5 consecutive days, followed by 2 days’ rest, for 4 weeks	HT7, SP6, GV20, KI6, BL62	2,6,7, TST, SOL, SE, WASO, N1, N2, N3 and REM^c^
Id.	45	Medication: 1–2 mg estazolam (tablet)	4 weeks					
Wu et al. (2021) (19)	32	Sham electroacupuncture	Once every other day, three times a week, for 4 weeks	34	Electroacupuncture	Once every other day, three times a week, for 4 weeks	① HT7, GV20, GV29, SP6 ② BL13, BL15, BL18, BL20, BL23	1,8,9
Wu et al. (2021) (20)	29	Medication: 1 mg of estazolam tablets	Every night for 4 weeks	30	Electroacupuncture	Once every other day, three times a week for 4 weeks	HT7, GV20, GV24, GV29, SP6	1,8, Serum GABA
Cao et al. (2023) (22)	41	Sham electroacupuncture	5 times a week for 5 weeks	44	Electroacupuncture	5 times a week for 5 weeks	HT7, GV20, SP6	1,7, Athens Insomnia Scale
Id.				43	Electroacupuncture	5 times a week for 5 weeks	HT7	1,7, Athens Insomnia Scale
Ding et al. (2023) (23)	30	Medication: estazolam (1 mg)	Every day for 20 days	30	Manual acupuncture	Every day for 20 days	HT7, GV20, SP6, KI6, BL62, Anmian	1, TCM syndrome integral
Huo et al. (2023) (24)	30	Sham acupuncture	Once every other day, 3 times per week for 4 weeks	30	Manual acupuncture	Once every other day, 3 times per week for 4 weeks	HT7, PC6, GV20, GV24, SP6, GB13, Sishencong	1,9,10, DST, TMT
Liu et al. (2024) (26)	48	Medication: 1 mg of lorazepam tablets	Every night for 4 weeks.	49	Manual acupuncture	5 consecutive days a week for 4 weeks.	GV20, HT7, SP6, BL62, KI5, BL15, BL23	1,7, Stroop Color-Word Test, Stroop Interference Effect
Zhang et al. (2024) (27)	53	Sham acupuncture	Once every other day for 4 weeks, with a total of 14 times.	51	Manual acupuncture	Once every other day for 4 weeks, with a total of 14 times.	BL15, BL20, HT7, SP6, BL62, KI5, ST36, Anmian	1,9, Hyperarousal scale, Heart Rate Variability
Guan et al. (2019) (28)	39	Medication: Estazolam (1–2 mg)	Every night for 4 weeks	40	Manual acupuncture	Treated 3 times a week for 4 weeks.	RN12, RN10, RN6, RN4, Sishencong, GV29, GV20, GV24	1,2,4
Wang et al. (2019) (29)	60	Medication: Estazolam (1 mg)	Every night for 4 weeks continuously	60	Manual acupuncture	Treated every day for 4 weeks.	GV20, HT7, SP6, BL62, KI6, Anmian (EX_HN)	1,4
Id.				60	Yinyang Ruyin manual acupuncture	Treated every day for 4 weeks.	DU20, PC6, SJ5, BL21, RN12, KI6	1,4
Zhang et al. (2019) (30)	90	Medication: Estazolam (1 mg)	Every night for 3 weeks continuously	90	Manual acupuncture	Treated every day for 3 weeks.	GV 20, Sishencong, Anmian, PC 6, HT 7, ST 36, SP 6, LR 2 and LR 3	1, TST, waking time, and non-rapid eye movement and REM
Yeung et al. (2009) (31)	30	Sham electroacupuncture	3 time per week for 3 weeks	30	Electroacupuncture	3 time per week for 3 weeks	Yintang (EX-HN3), GV20, bilateral ear Shenmen, Sishencong, and Anmian	1,2,11, SOL, WASO, TST, SE, Sheehan Disability Index, the Credibility of Treatment Rating Scale
Wang et al. (2021) (32)	29	Sham electroacupuncture	Once every other day, 3 times per week for 4 weeks	30	Electroacupuncture	Once every other day, 3 times per week for 4 weeks	HT7, DU20, SP6, GV29, BL15, BL23	1, 10, Serum concentrations of MT and DA
Yu LJ et al. (2024) (34)	34	Medication: Estazolam (1 mg)	Every night for 4 weeks continuously	33	Electroacupuncture	6 consecutive days a week for 4 weeks	GB20, Gongxuexue, GV20, LR3, SP6	1, Auditory Verbal Memory Test (AVMT), TCM syndrome integral, IL-1β, IL-6, IL-8
Liu L et al. (2025) (33)	29	Medication: Estazolam (1 mg)	Every night for 4 weeks continuously	29	Manual acupuncture	6 consecutive days a week for 4 weeks	GB20, GB13, HT7, SP6	1, PSG, 5-HT, DA

a 1- Pittsburgh Sleep Quality Index score (PSQI), 2- Insomnia Severity Index (ISI), 3- Self-Rating Depression Scale (SDS), 4- Total effective rate, 5- Epworth Sleepiness Scale (ESS), 6- Auditory Verbal Memory Test (AVMT), 7- Polysomnography (PSG), 8- Cortisol (CORT) levels, 9- Fatigue Scale-14 (FS-14), 10- Montreal Cognitive Assessment (MoCA), 11- Hospital Anxiety and Depression Scales (HADS).

b NA, not applicable.

c TST, total sleep time; SOL, sleep onset latency; WASO, wake after sleep onset; SE, sleep efficiency; N1, N2, N3: non-rapid eye movement period; and REM, rapid eye movement period.

### 2.4 Risk of bias assessment

This assessment was performed by the two reviewers (Peiqi Li and Qianwen Yang) independently using version 2 of the Cochrane tool for assessing the Risk of Bias (RoB2) in randomized trials. The randomization process, deviations from intended interventions, missing outcome data, outcome measurement, and selection of reported results were the five factors used to assess the risk of bias.

All the studies were classified into “low risk of bias,” or “high risk of bias,” or “unclear.” Possible disagreements were settled by the third reviewer (Qinhui Fu).

### 2.5 Data synthesis

Review Manager Software (RevMan, version 5.4) was utilized for all statistical analyses. The primary outcomes were the PSQI and ISI scores. To adhere to the Cochrane unit-of-analysis guidance, we used only one pair of comparisons to conduct the pairwise meta-analysis. The preliminary input rules were as follows: (1) when there were two kinds of experiment intervention, we would choose single acupuncture or usual acupuncture rather than acupuncture with special manipulations or acupoints, introducing less heterogeneity, and (2) when there were two kinds of control intervention, we would primarily choose drugs rather than sham acupuncture or waiting list, in order to better present the difference between acupuncture and the conventional pharmacological therapy. As for secondary outcomes, four neurobehavioral assessment tools were selected to evaluate different daytime symptoms: (1) Epworth Sleepiness Scale (ESS) is used to quantifying drowsiness tendencies in different daytime situations, (2) Auditory Verbal Memory Test (AVMT) could measure four hippocampal-dependent memory consolidation critical for daytime cognitive performance, (3) Montreal Cognitive Assessment (MoCA), recognized for its high diagnostic sensitivity and clinical feasibility, serves as a gold standard brief cognitive screening tool across for overall cognitive function, and (4) Fatigue Scale-14 (FS-14) is chosen to quantify the severity of fatigue, indirectly reflecting the quality and recovery effect of sleep at night. Other outcomes, such as cortisol (CORT) level and total effective rate, were also recorded if reported. Quantitative data, such as sample size, were extracted from all the eligible RCTs, as well as means and standard deviation values of outcome measurements (PSQI and ISI scores) after treatment in each group. We exclusively extracted data from the earliest post-treatment time-point (usually immediately after the completion of the treatment intervention). If a study reported results of multiple post-treatment or follow-up time-points, data from the earliest one were prioritized. Standardized mean difference (SMD), mean difference (MD), and 95% confidence intervals (CIs) were used for the efficacy analysis of continuous variables.

Note that when there were no direct data on mean value and standard deviation, we calculated them through the size, first quartile, median, and third quartile of the sample using a statistical formula (15, 16).

The statistical heterogeneity of the results was evaluated using the *I*^2^ test, and then a fixed or random-effect model would be applied accordingly. Additional analyses (e.g., sensitivity analysis and subgroup analysis) were executed to identify potential sources of heterogeneity and assess the suitability of using a random-effects model. Furthermore, evaluations of subgroups were carried out to look into heterogeneity pertaining to the intervention, treatment duration, number of acupoints used, treatment frequency, diagnostic criteria, age range, and article quality. Two methods were used to test the interactions between the factors: (1) qualitative assessment: an interaction was probably absent if each subgroup’s 95% CI overlapped; and (2) quantitative assessment: an interaction was probably absent if the subgroup difference was greater than 0.05.

In addition, funnel plots were employed to evaluate potential bias in the RCTs encompassed in this review when the total number of trials included was more than or equal to 10. When heterogeneity larger than 50% was referred to a random-effects model application, the RevMan software was unable to generate funnel plots with 95% CI lines. Therefore, Stata 13.1 software was utilized to generate the corresponding funnel plot. The publication bias was deemed low if both sides of the funnel plots were balanced.

### 2.6 Quality of evidence assessment

The quality of evidence was assessed and classified into four levels: high, moderate, low, and very low quality, according to the Grading of Recommendations Assessment, Development, and Evaluation (GRADE). The evidence quality was assessed separately by two authors using the Guideline Development Tool (GRADEpro GDT). Disagreements were settled through additional discussion.

## 3 Results

### 3.1 Literature retrieval

**Figure 1:**
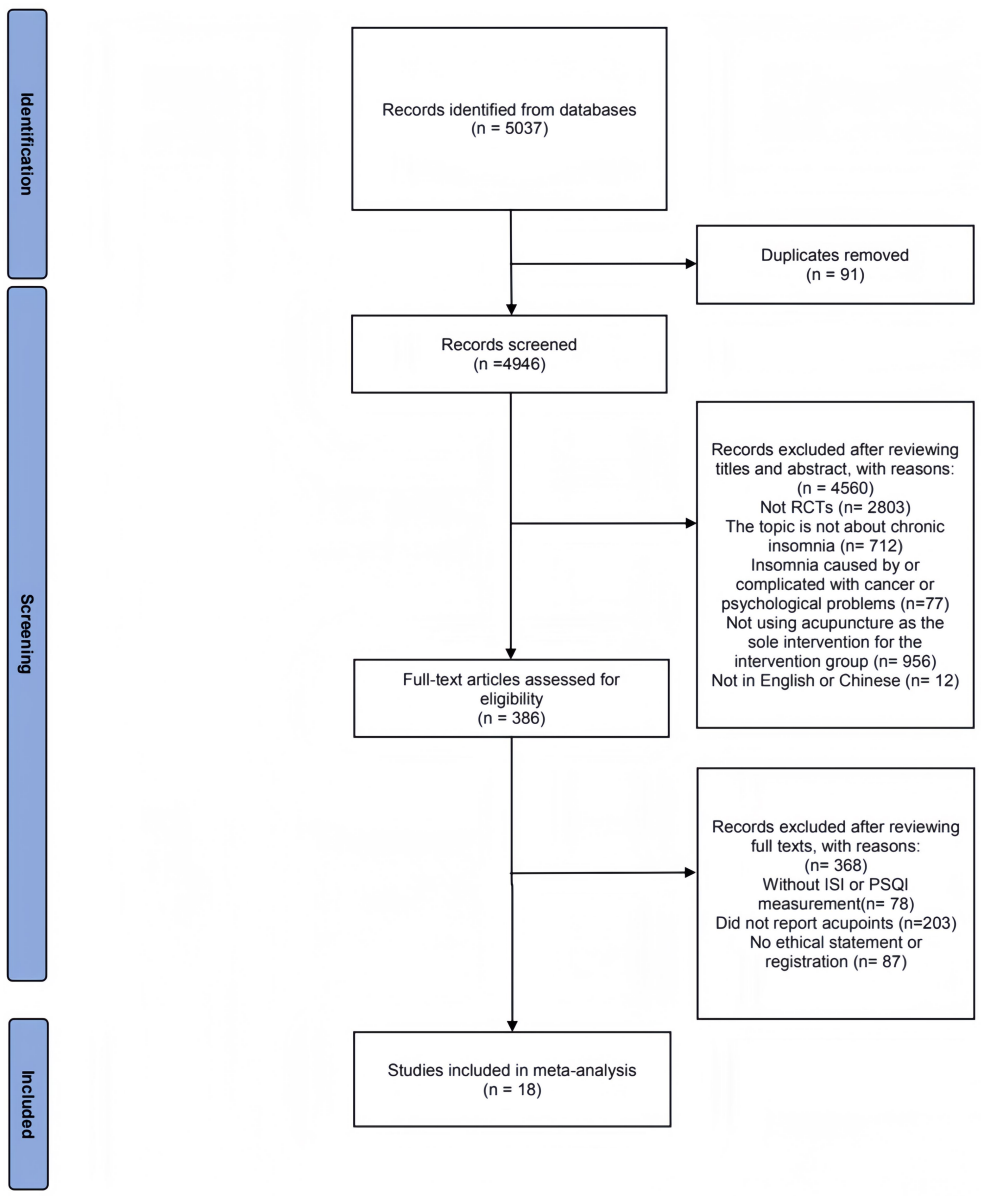
Flowchart illustrating the process of literature retrieval.

In Figure 1, the diagram illustrates that 5,037 studies were found across the six databases. Ninety-one replications were removed, and no grey literature was added. Based on titles, keywords, and abstracts, 4,560 articles were excluded, including reviews, case reports and series, protocols, animal experiments, letters, surveys, and commentaries. The articles that did not focus on insomnia were excluded (*n* = 712). In the remaining articles, participants with insomnia caused or complicated by cancer or psychological problems (*n* = 77), studies that did not use acupuncture as the exclusive treatment for the intervention group (*n* = 956), and articles not published in English or Chinese (*n* = 12) were excluded. The complete texts of the remaining 386 studies were acquired and subsequently evaluated; 368 studies were eliminated due to the specified reasons: no ISI or PSQI measurements (*n* = 78), did not report acupoints used (*n* = 203), and no ethical statement or registration was reported (*n* = 87). Finally, 18 randomized controlled trials that satisfied the criteria for inclusion were ultimately incorporated into the meta-analysis.

### 3.2 Article characteristics

Totally, 1,767 patients were included from the 18 RCTs (17–35). Four of the studies were published in English, and 14 in Chinese. Participants were identified as having insomnia based on either the third edition of the International Classification of Sleep Disorders (ICSD-3) or the fifth edition of the Diagnostic and Statistical Manual of Mental Disorders (DSM-5). In each study, the participants’ characteristics (age, sex, and other parameters) at baseline were balanced between the treatment and control groups (Table 2). The interventions were manual acupuncture in 11 studies and electroacupuncture in the other 7 studies. The comparators included no treatment (waiting list), sham (electro-) acupuncture, and medication. In these trials, the course of treatment varied from 2 to 5 weeks, and each treatment lasted approximately 30 min. The treatment frequency of acupuncture and electroacupuncture was either once a day or once every other day, while that of drug therapy was generally every night.

### 3.3 Quality evaluation

**Figure 2:**
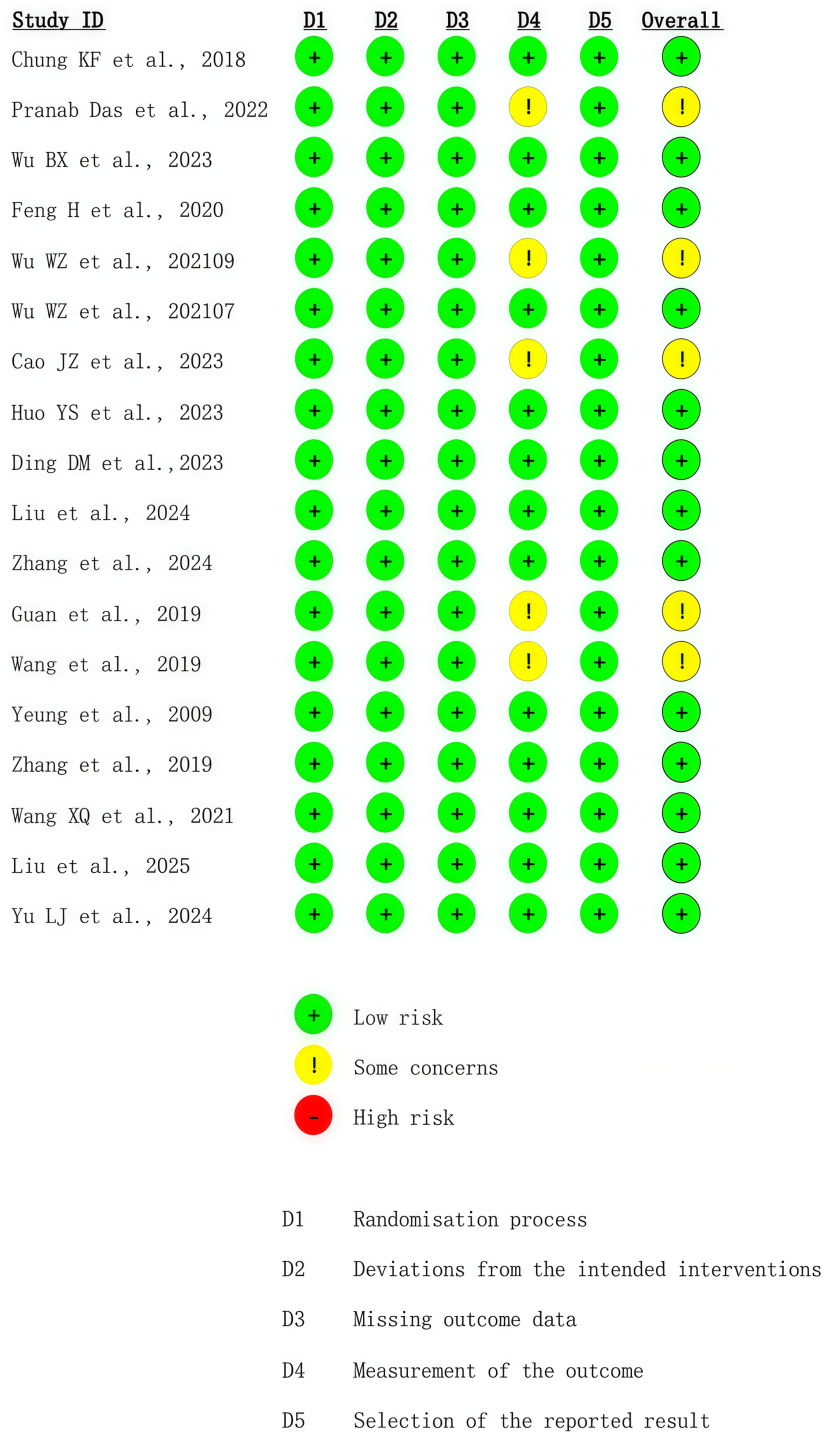
Results of the RoB2 assessment.

**Figure 3:**
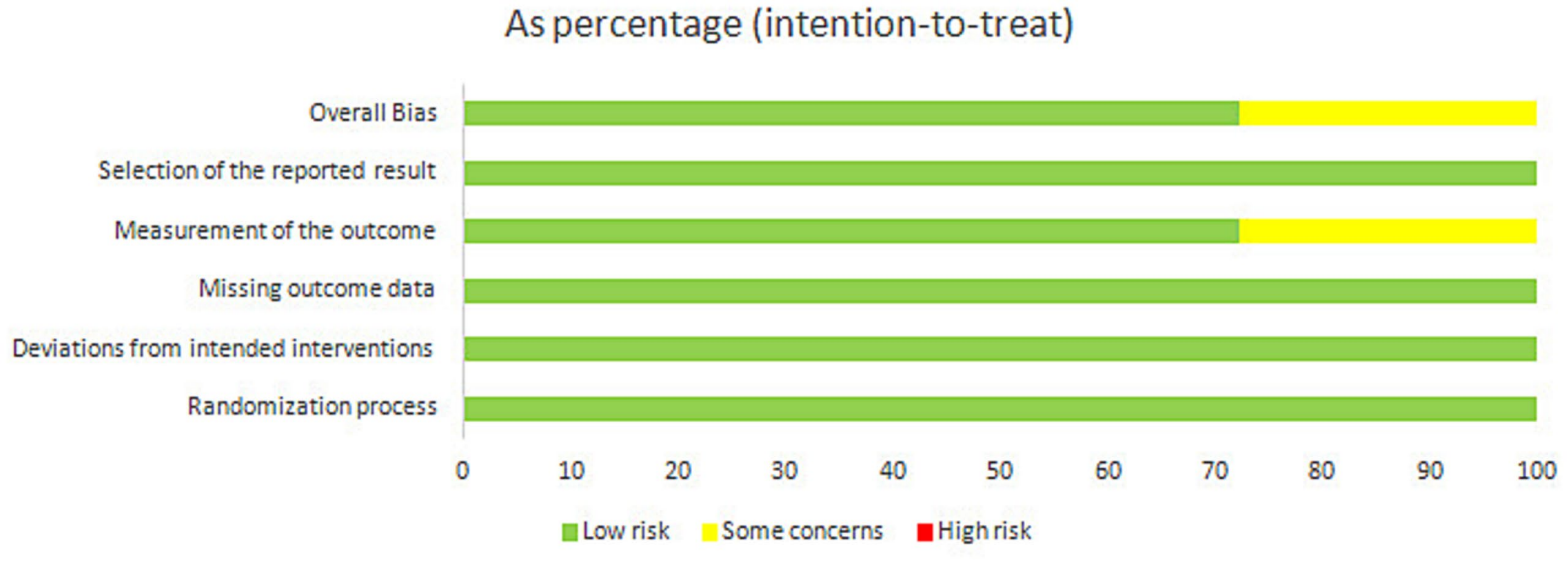
Risk of bias diagram.

Randomization was applied in all 18 included studies. The results showed a low risk of deviation from the intended interventions. The lack of missing outcome data degrades the risk of bias. Regarding outcome measurements, five studies raised concerns about missing outcome data owing to dropouts. Almost all trials blinded the outcome assessors to the participants’ interventions. The risk of selection of the reported results was low. The results of the risk of bias assessment for all included studies are illustrated in Figures 2, 3. In general, 13 studies were of low risk, whereas 5 exhibited some concern.

### 3.4 Results of main outcome measurements

#### 3.4.1 Result of meta-analysis of PSQI total scores

**Figure 4:**
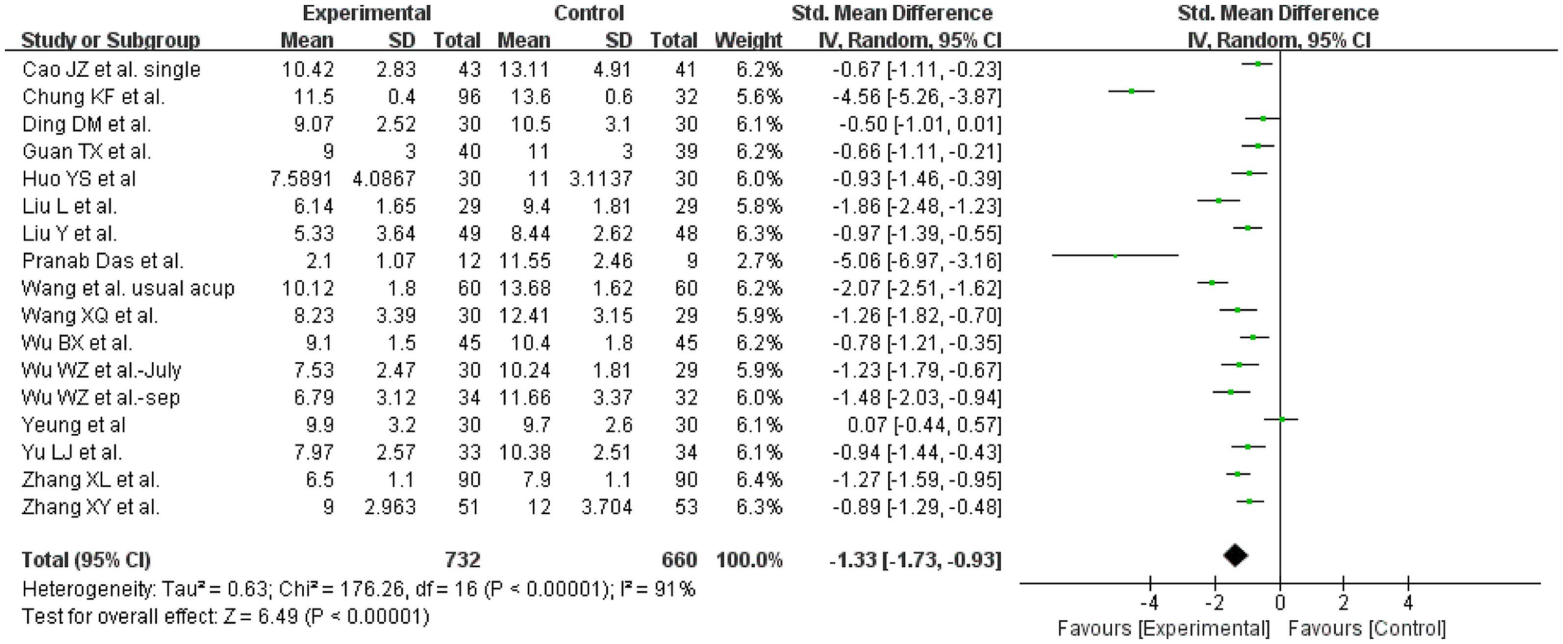
Synthetic data of PSQI total scores.

The PSQI was used to assess insomnia in 17 studies. Totally, 1,392 patients comprising 732 in the experimental group and 660 in the control group were sorted (Figure 4). The heterogeneity result was *p* < 0.05, *I*^2^ = 91%. Thus, a random-effects model was administered to calculate the synthesized std. mean difference. The meta-analysis revealed that acupuncture can significantly improve the PSQI global score when assessed by the PSQI scores after treatment (SMD = -1.33, 95% CI:[−1.73, −1.93], *p* < 0.05) (Figure 4). The result of the Begg’s test was Pr > |z| = 0.039 < 0.05, while that of the Egger’s test was *P* > |t| = 0.058 < 0.05. These calculations suggest the presence of publication bias, and the results should be interpreted with cautious.

#### 3.4.2 Outcome of PSQI subscale scores

Eleven articles reported scores of PSQI subscales, including the subjective sleep quality, sleep latency, sleep duration, and sleep disturbances. In total, there were 421 patients in the acupuncture group and 418 in the control group.

**Figure 5:**
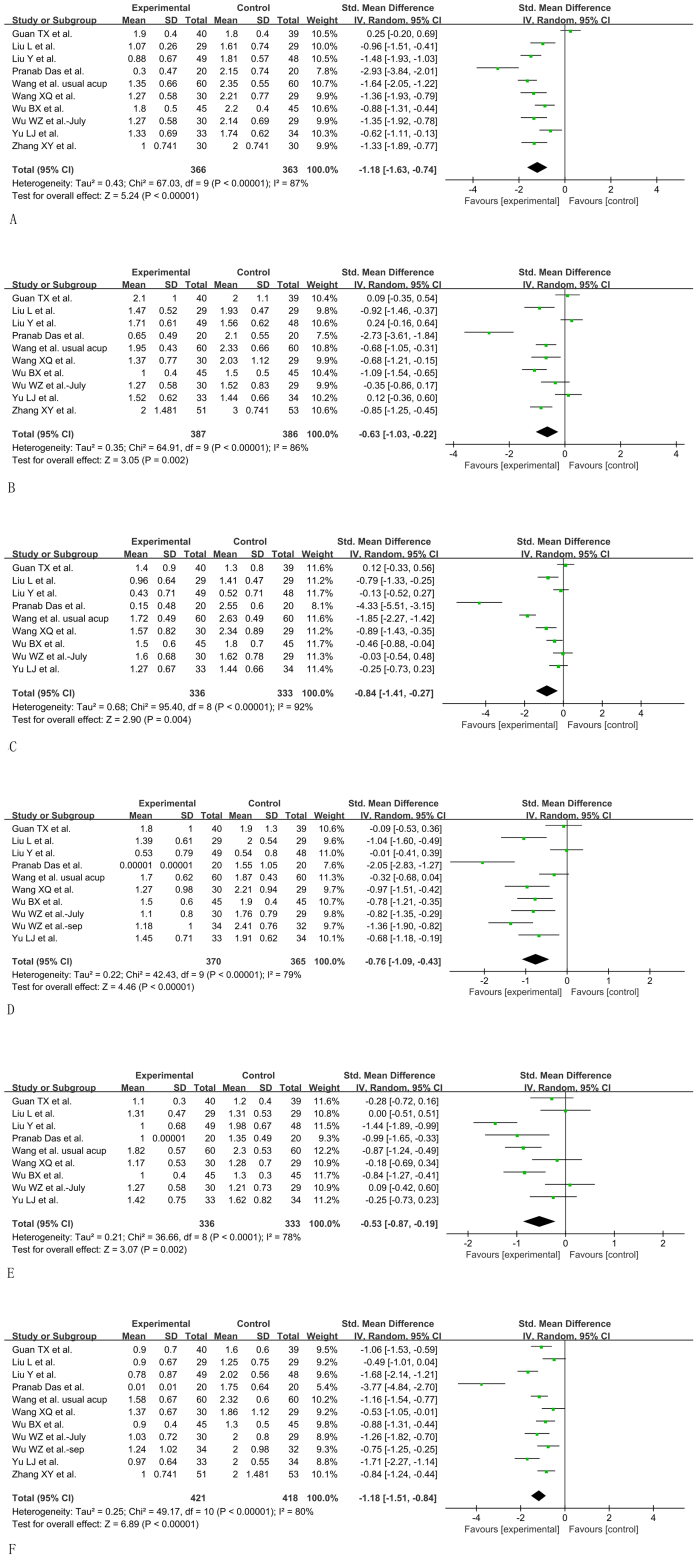
Synthetic data of PSQI subscale scores. **(A)** Subjective sleep quality. **(B)** Sleep latency. **(C)** Sleep duration. **(D)** Habitual sleep efficiency. **(E)** Sleep disturbance. **(F)** Daytime dysfunction.

When synthesized by random-effects, the result illustrated that acupuncture could significantly:

- Improve subjective sleep quality (SMD = −1.18, 95%CI: −1.63 to −0.74, *p* < 0.05, I^2^ = 87%) (Figure 5A).
- Decrease sleep latency (SMD = −0.63, 95%CI: −1.03 to −0.22, *p* < 0.05, I^2^ = 86%) (Figure 5B).
- Prolong sleep duration (SMD = −0.84, 95%CI: −1.41 to −0.27, *p* < 0.05, I^2^ = 92%) (Figure 5C).
- Improve habitual sleep efficiency (SMD = −0.76, 95CI%: −1.09 to −0.43, *p* < 0.05, I^2^ = 79%) (Figure 5D).
- Reduce sleep disturbance (SMD = −0.53, 95CI%: −0.87 to −0.19, *p* < 0.05, I^2^ = 78%) (Figure 5E).
- Reduce daytime dysfunction (SMD = −1.18, 95CI%: −1.51 to −0.84, *p* < 0.05, I^2^ = 80%) (Figure 5F).

#### 3.4.3 Outcome of synthesized ISI scores

**Figure 6:**
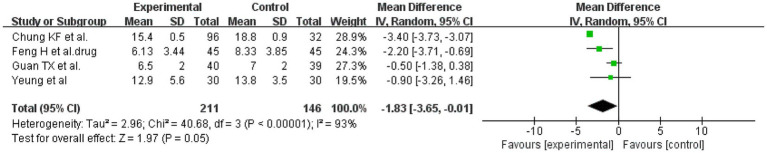
Synthesized result of ISI scores.

Four studies reported ISI scores. Totally, 211 patients were treated by acupuncture, and 146 were in the non-acupuncture group. Substantial heterogeneity existed (*p <* 0.05, *I*^2^ = 93%); therefore, a random-effects model was applied. The pooled result indicated that acupuncture has a significant effect compared to control interventions (MD = −1.83, 95% CI: −3.65 to −0.01, *p* = 0.05) (Figure 6) in relieving insomnia.

### 3.5 Exploration of the source of heterogeneity

#### 3.5.1 Analyzing the heterogeneity among PSQI total scores

To assess the stability of the results, we ran a sensitivity analysis in which one study was removed each time, and the pooled estimate was calculated again for the rest of the included studies. When removing the article written by Chung KF et al. (17), the heterogeneity reduced a little, from 91 to 81%. The results of the included studies were all positive and set on the same side of the forest plot. Therefore, the different levels of effectiveness may be the main source of heterogeneity. The results of the influence analysis of PSQI scores implied that the effect of each individual study on the pooled estimate was relatively small, and in general, the results of our study were relatively robust and credible.

Subgroup analyses were conducted with respect to intervention and gender distribution. However, the heterogeneity of these subgroups was greater than 50%, so these factors were not the source of heterogeneity. In addition, we used Stata 13.1 to manage the meta-regression and further analyze the confounding factors. Meta regression analysis (Table 3) proved no significant correlations between the pooled therapeutic effects and covariate factors, such as control intervention, experimental intervention, number of acupoints used, treatment frequency, gender distribution, age range, or article quality. Only when the articles were sorted according to different diagnostic criteria did one of the subgroup heterogeneities decrease to about 16.8%, identifying this factor as the source of between-study variability. Additionally, the meta regression analysis revealed a correlation between the treatment duration and the pooled result (*p* < 0.05). By sorting the included articles according to different treatment durations ranging from 2 to 5 weeks, the heterogeneity could be reduced. After sorting and further calculation, each pooled effect was still statistically significant, meaning stable positive results.

**Table 3:** Results of subgroup analysis and meta-regression.

Covariate factors	Std. Err.	*p*	95% CI-L	95% CI-U	Subgroup heterogeneity
Control intervention	0.4301007	0.21	−1.479562	0.3539144	>79.3%
Experimental intervention	0.636543	0.88	−1.454249	1.25927	>85.2%
Treatment duration	0.4335937	0.005	0.508928	2.357294	>78.8%
Number of acupoints used	0.6634194	0.943	−1.462619	1.365471	>81.5%
Treatment frequency	0.6445849	0.92	−1.307713	1.440087	>84.4%
Gender distribution	0.4753323	0.291	−0.4934108	1.532883	>90.3%
Diagnostic criteria	0.3141569	0.132	−1.169625	0.1695944	>16.8%
Age range	0.3150388	0.077	−0.0726495	1.270329	>81.7%
Article quality	0.7063342	0.699	−1.784201	1.22683	>85.7%

In summary, the diagnostic criteria and the treatment duration were the two main sources of heterogeneity, even though they would not affect the general meta-evidence that acupuncture was significantly effective for enhancing the PSQI total score.

#### 3.5.2 Sensitivity analysis of PSQI subscale scores

Each article was extracted individually to enable the calculation of heterogeneity and the effective size of the remaining studies to conduct sensitivity analysis.

When Guan et al.’ s (28) article was removed, the heterogeneity among the scores of subjective sleep quality decreased from 87 to 71%, and the overall effect was still statistically significant, meaning firm positive results support acupuncture’s therapeutic effects. The control intervention caused the heterogeneity in this study. This study was the only one that allowed patients to take more than 1 mg of estazolam, while in other studies, only 1 mg of estazolam per night was utilized as the control intervention.

For the subscale scores of sleep latency, sleep duration, and habitual sleep efficiency, sensitivity analysis could only decrease about 5% of the heterogeneity. The major heterogeneity came from the extremely effective report from the article by Pranab Das (21).

The source of heterogeneity in the sleep disturbances subscale was caused by the outstanding effectiveness of Liu Y’s article (26), and the I^2^ could be decreased to 65% after removing this article.

In the aspect of the daytime dysfunction subscale, the heterogeneity was caused by the outstanding effectiveness of Pranab Das et al.’s article (21), and the I^2^ could be decreased to 63% after removing this article. However, the synthesized result was still concrete that acupuncture was effective for improving daytime function.

#### 3.5.3 Exploring the source of heterogeneity in the ISI data

In the case of the substantial heterogeneity (*I*^2^ = 93%) of ISI scores, sensitivity analyses were performed to determine the source of robustness of the synthesized outcomes. After removing the outstanding favorable data of the study by Chung et al. (17), the heterogeneity decreased to 45%. However, the statistical significance of the pool effect size was lost (*p* = 0.06), indicating that the current evidence is limited. Therefore, caution is warranted in interpreting these results, and future research with larger sample sizes is needed.

### 3.6 Secondary outcome syntheses

When enough data of the same measurement were reported in more than one study, they were extracted and are shown in Table 4. Meta-calculations were then performed using RevMan 5.4 software.

**Table 4:** Summary of secondary outcomes.

Outcome measurement	Study	Statistical results reported in the article	Results of meta calculation
ESS	Chung et al. (2018) (17)	*p* < 0.05	NA
	Das et al. (2022) (21)	*p* < 0.05	
AVMT	Wu et al. (2023) (25)	*p* < 0.05	Instant memory: I^2^ = 87%, *p* < 0.05, MD [95% CI]: 4.65 [2.25, 7.06] Short delayed-recall: I^2^ = 0%, *p* < 0.05, MD [95% CI]: 1.35 [1.01, 1.69] Long delayed-recall: I^2^ = 0%, *p* < 0.05, MD [95% CI]: 1.14 [0.78, 1.50] Delayed recognition: I^2^ = 70%, *p* < 0.05, MD [95% CI]: 2.55 [1.58, 3.52]
	Feng et al. (2020) (18)	*p* < 0.05	
	Yu LJ et al. (2024) (34)	*p* < 0.05	
CORT	Wu et al. (2021) (19)	*p* > 0.05	NA
	Wu et al. (2021) (20)	*p* < 0.05	
FS-14	Huo et al. (2023) (24)	*p* < 0.05	I^2^ = 89%, *p* < 0.05, MD [95% CI]: −3.00 [−4.69, −1.32]
	Wu et al. (2021) (19)	*p* < 0.05	
	Zhang et al. (2024) (27)	*p* < 0.05	
Total effective rate	Guan et al. (2019) (28)	*p* > 0.05	I^2^ = 85%, *p* < 0.05, OR [95% CI]: 2.83 [1.79, 4.48]
	Wang et al. (2019) (29)	*p* < 0.05	
	Zhang XL et al. (2019) (30)	*p* < 0.05	
	Yu LJ et al. (2024) (34)	*p* < 0.05	
MoCA	Huo et al. (2023) (24)	*p* < 0.05	I^2^ = 0%, *p* < 0.05, MD [95% CI]: 1.37 [1.01, 1.73]
	Wu et al. (2023) (25)	*p* < 0.05	
	Wang XQ et al. (2021) (32)	*p* < 0.05	

ESS, Epworth sleepiness scale; AVMT, auditory verbal memory test; CORT, cortisol level; FS-14, Fatigue Scale-14; MoCA, Montreal Cognitive Assessment; MD, mean difference; CI, confidence interval; OR, odds ratio.

The synthesized data showed that acupuncture therapy had significant curative effects when measured using four subscales of AVMT (instant memory, delayed recognition, short delayed recall, and long delayed recall), FS-14, total effective rate, and MoCA. Nevertheless, the heterogeneity of some results was generally high, so these results should be interpreted cautiously.

Original data of CORT and ESS scores were not reported in the included articles, preventing a meta-analysis. Accordingly, we extracted the reported *p*-values and summarized them in the table above to provide a general overview.

### 3.7 Adverse effects

Among the included studies, seven studies reported negative events and the exact number of patients who experienced them (see Table 5). The most common event was a subcutaneous hematoma, predominantly in the acupuncture intervention groups. Drug-related effects emerged, such as thirst and lethargy, in the medication control groups. The majority of events were mild to moderate in severity, indicating a favorable overall safety profile for the interventions studied. In these studies, there were no withdrawals from the study owing to adverse effects.

**Table 5:** Adverse effects reported.

Intervention of control group	Studies	Control group	Treatment group
Sham(electro-) acupuncture	Feng et al. (2020) (18)	2 (subcutaneous hematoma and fainted during acupuncture treatment)	1 (subcutaneous hematoma)
	Wu et al. (2021) (19)	1 (fatigue)	2 (1 subcutaneous hematoma, and 1 regional pain)
	Cao et al. (2023) (22)	3 (abnormal feeling after acupuncture)	10 (6 abnormal feeling after acupuncture, and 4 subcutaneous hematoma)
	Yeung et al. (2009) (31)	3 (1 headache, 1 hand numbness, 1 worsening of insomnia)	4 (2 headache, 1 subject developed hand numbness, and 1 subcutaneous hematoma)
Medication	Wu et al. (2023) (25)	2 (thirst)	3 (subcutaneous hematoma)
	Feng et al. (2020) (18)	2 (headache and lethargy)	1 (subcutaneous hematoma)
	Ding et al. (2023) (23)	1 (drug dependence)	1 (fainted during acupuncture treatment)
	Yu LJ et al. (2024) (34)	2 (headache and sicchasia)	3 (subcutaneous hematoma)

Other than the above six studies, Chung et al. (17) also reported the adverse events, but without the exact number of each incident. The study reported that the total number of adverse events was 260, of which 243 were mild, 14 moderate, and 3 severe, with the incidence rate of any negative event being 62%. The top three adverse events were acupoint bruising (26.6%), headache and other pain events (21.4%), and needle site pain (17.7%). Moreover, 2.1 and 3.6% of the participants in the acupuncture treatment group and combination therapy groups, respectively, dropped out because of adverse events. The authors of that study emphasized that all reported events were mild and the treatments were deemed safe and acceptable to patients.

### 3.8 Overall quality of outcome evidences

**Figure 7:**
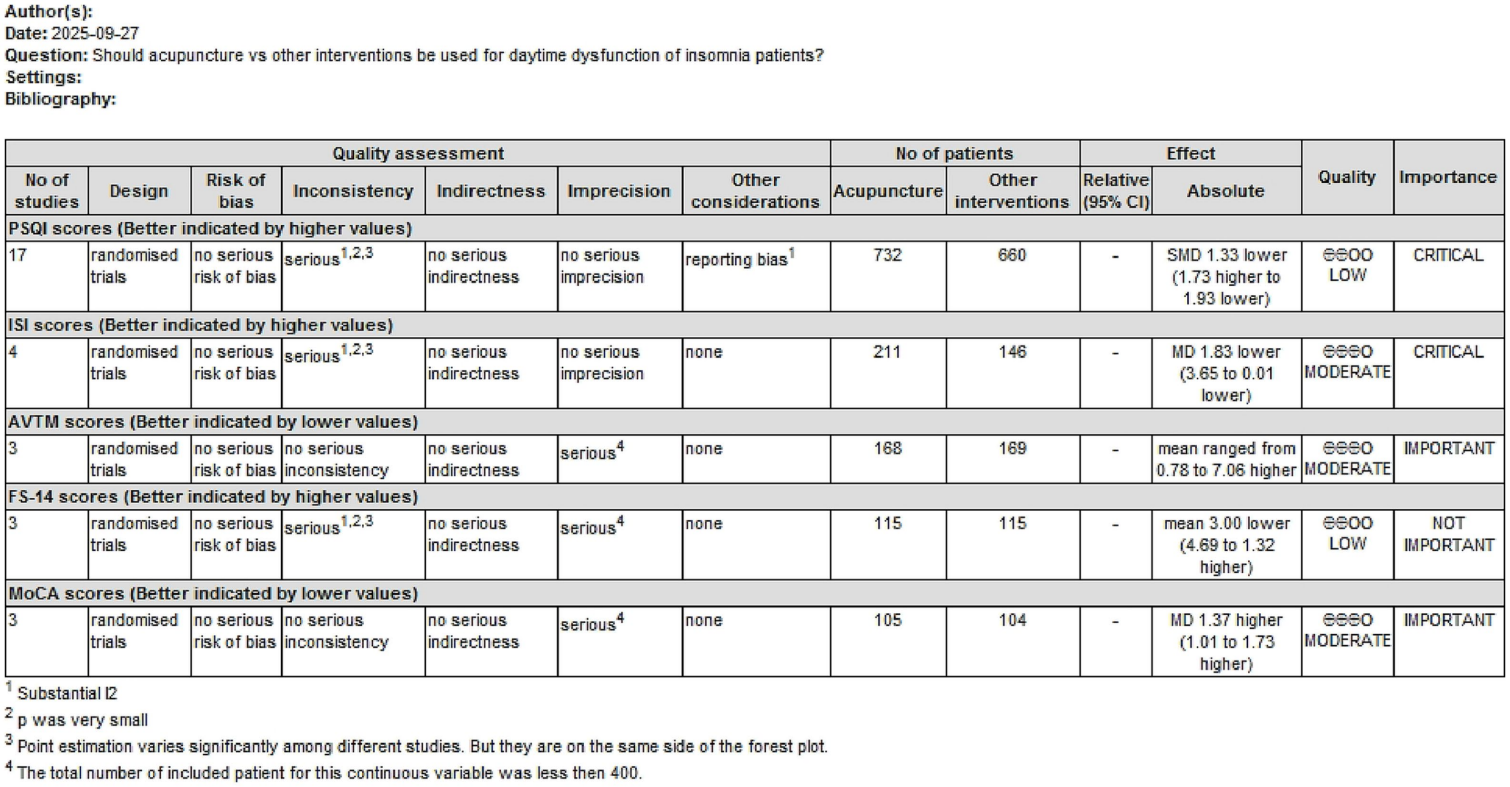
Summary of findings (SoF) generated by GRADE.

The evidence for the quality of the synthesized results was assessed by the GRADE process (Figure 7). Evidence of moderate quality supported that acupuncture could improve ISI, AVMT, and MoCA scores. In contrast, the evidence of acupuncture improving PSQI and FS-14 scores was of low quality.

## 4 Discussion

Our meta-analysis encompassing 1,767 patients showed that acupuncture could decrease PSQI scores and ISI scores, which meant an improvement in insomnia symptoms. This coincides with previous meta-analyses (11–13) that acupuncture could improve sleep quality, sleep initiation, sleep stability, and sleep efficiency, thereby reducing the general insomnia severity.

The daytime function of insomnia patients was measured by four indexes: AVMT, MoCA, FS-14, and ESS. The enhancement of these scores suggests that acupuncture functions not merely as a simple intervention for difficulty falling asleep but as a holistic therapy that addresses the interconnected cluster of daytime issues. The observed benefits may be linked to its multi-pathway regulatory effects. Our synthesized results of AVMT implied that acupuncture potentially improves sleep-dependent memory processes (36), a notion supported by clinical evidence showing acupuncture can enhance memory performance in insomnia patients alongside increasing the proportion of N3 (slow-wave) sleep (37), which is critical for memory consolidation (38). The improvement in general cognitive state measured by the MoCA scale may be attributed to its potential to modulate key neurotransmitters and hormones. Preliminary evidence suggests it may influence gamma-aminobutyric acid (GABA) level (39) and cortisol rhythm (20). On one hand, acupuncture could elevate GABA levels and modulate GABA receptor activity (40–42), thereby stabilizing the non-rapid eye movement (NREM) sleep. More specifically, acupuncture prolongs the N3 stage of sleep (2, 37), which theoretically could extend the temporal window for glymphatic system activity (43–45). The glymphatic system facilitates the exchange of cerebrospinal fluid (CSF) with interstitial fluid within the brain parenchyma (46) and enhances intracranial substance metabolism. This theoretically enhanced brain-waste-clearance process is hypothesized to contribute to the improved neural function and cognition, including memory. On the other hand, acupuncture could help adjust the cortisol level alignment with the sleep–wake cycle (47, 48). By lowering nocturnal cortisol levels, acupuncture may potentially reduce suppression of the hippocampus and prefrontal cortex, supporting cognitive functions, such as memory, attention, decision-making, and emotional regulation (49, 50).

Moreover, our results of decreased FS-14 scores implied that acupuncture could reduce insomnia-related fatigue. The possible pathways may be that acupuncture could increase cortisol levels in the daytime (49, 50), leading to a lesser sense of fatigue (51). Acupuncture could inhibit the high protein expression levels of pro-inflammatory factors IL-1β, IL-6, and TNF-*α* in the hippocampus, which occurred under the fatigue condition (52), thereby improving the microenvironment of the brain and relieving fatigue symptoms. With less emergence and more relief of fatigue, patients’ daytime performance could be improved.

Additionally, our systematic review of ESS scores implied that acupuncture could reduce drowsiness tendency in the daytime, which is similar to the previous literature (14). A potential mechanism involves the adjustment of serotonin (5-HT), a key neurotransmitter that adjusts the cycles of alertness and sleepiness (53). Acupuncture could increase the 5-HT level in the daytime (33) so that the alertness is increased and the drowsiness is reduced, bringing a clearer mind state to insomnia patients.

To sum, acupuncture could improve sleep quality and daytime dysfunction at the same time, regardless of the type of acupuncture, or patients’ gender or age. This positions acupuncture as a suitable intervention for a broad range of patients, including specific populations such as athletes, perimenopausal women, and older adults, who seek enhanced daytime cognition and neural function. Acupuncture therapies are more feasible and accessible even in the community, so we hope these insights advocate for integrating acupuncture into clinical practices, offering a promising non-pharmacological approach to personalized management of insomnia and its related comorbidities.

While this study provides moderate-quality evidence supporting acupuncture for insomnia-related daytime dysfunction, several limitations warrant consideration and frame future research directions. First, the heterogeneity of the included studies is inevitable, reflecting diversity in real-world acupuncture protocols (e.g., diagnostic criteria, acupoint selection, stimulation techniques, and treatment frequency) and in the studied insomnia populations themselves. Stratifying studies by diagnostic criteria (e.g., DSM-5, ICSD-3) could substantially reduce heterogeneity. This indicates that variability in treatment response is partly attributable to diagnostic precision, with stricter criteria defining a more homogeneous cohort. Consequently, clinicians must consider individual patient characteristics alongside the overall positive effect when making clinical decisions.

Second, the generalizability of our findings is constrained by the predominance of Chinese trials and the limited duration of follow-up in the majority of the included trials. Therefore, future research is recommended to prioritize multi-center trials with larger, more diverse populations, longer follow-up periods, and standardized treatment and reporting protocols to confirm efficacy and enhance generalizability. Moreover, blinding challenges in acupuncture trials may also inflate effect estimates. Thus, it is encouraged to apply rigorous assessment of patient blinding efficacy to disentangle genuine therapeutic effects from placebo responses.

The above mechanistic pathways discussed, while plausible and informed by existing literature, remain preliminary and require validation through integrated neurobiological and clinical studies. Furthermore, mechanistic insights would be strengthened by integrating objective outcome measurements, including glymphatic imaging, functional magnetic resonance imaging (fMRI), functional near-infrared spectroscopy (fNIRS), and molecular proxies of metabolic waste clearance. It would also be valuable for future studies to situate acupuncture within the broader landscape of non-pharmacological neuromodulation by including comparative effectiveness research with other emerging interventions, such as transcutaneous auricular vagus nerve stimulation (54), which has also shown promise in improving sleep scales. Finally, the cost-effectiveness of acupuncture therapies versus other therapies deserves further research.

## 5 Conclusion

This meta-analysis positions acupuncture as a multifaceted intervention for chronic primary insomnia, suggesting potential benefits for both nocturnal disturbances and daytime impairment. The measurements of daytime function, memory, cognition, and nighttime sleep quality mutually corroborate acupuncture’s therapeutic role. The findings demonstrate consistency in directionality and effect magnitude. However, these findings must be interpreted with caution, given the low-to-moderate quality of some of the evidence.

## Data Availability

The original contributions presented in the study are included in the article/Supplementary material, further inquiries can be directed to the corresponding authors.

## Glossary

AVMT: auditory verbal memory test
CBT-i: Cognitive Behavioral Therapy for Insomnia
CENTRAL: Cochrane Central Register of Controlled Trials
CI: confidence interval
CORT: cortisol level
CSF: cerebrospinal fluid
DSM-5: the Diagnostic and Statistical Manual of Mental Disorders
ESS: Epworth sleepiness scale
FS-14: Fatigue Scale-14
GABA: gamma-aminobutyric acid
GRADE: Grading of Recommendations Assessment, Development and Evaluation
GRADEpro GDT: Guideline Development Tool
HPA: hypothalamic - pituitary - adrenal
ICSD-3: International Classification of Sleep Disorders
ISI: Insomnia Severity Index
MeSH: Medical Subject Headings
MMSE: mini-mental state examination
MoCA: Montreal Cognitive Assessment
MD: mean difference
NREM: non-rapid eye movement
OR: odd ratio
PSQI: Pittsburgh Sleep Quality Index
RCT: randomized controlled trial
RoB2: version 2 of the Cochrane tool for assessing the Risk of Bias
SMD: Standardized mean difference
SoF: Summary of Findings
TENS: transcutaneous electrical nerve stimulation
5-HT: serotonin
