# Supplementary material for: Beyond nighttime symptoms: acupuncture for daytime dysfunction improvement in insomnia—a meta-analysis
Source: Front Neurol. 2026 Mar 3;17:1752313. doi: 10.3389/fneur.2026.1752313 (PMC12992216; doi:10.3389/fneur.2026.1752313)
Supplement: Supplementary file 1 [file Table_1.DOCX]

**Supplementary Material 1**

Search strategy for Embase:

Input "(('acupuncture'/exp OR 'acupuncture point'/exp OR 'meridian'/exp OR 'electroacupuncture'/exp OR 'dry needling'/exp OR 'trigger point'/exp) OR (acupuncture:ti,ab OR 'acupuncture point*':ti,ab OR 'electroacupuncture':ti,ab OR 'electro-acupuncture':ti,ab OR 'dry needling':ti,ab OR 'trigger point*':ti,ab OR meridian:ti,ab OR 'body acupuncture':ti,ab OR 'coiling dragon':ti,ab OR 'dermal needle':ti,ab OR 'point injection':ti,ab)) AND (('insomnia'/exp OR 'sleep initiation and maintenance disorder'/exp) OR (insomnia:ti,ab OR 'chronic insomnia':ti,ab OR 'primary insomnia':ti,ab OR 'sleep initiation':ti,ab OR 'sleep maintenance':ti,ab OR 'early awakening':ti,ab OR sleeplessness:ti,ab OR 'sleep disorder*':ti,ab))" in "advanced search".

Search strategy for PubMed:

| Number | Search items |
| --- | --- |
| #1 | (((((("Acupuncture"[MeSH]) OR "Acupuncture Points" [MeSH]) OR "Acupuncture Therapy"[MeSH]) OR "Dry Needling"[MeSH]) OR "Trigger Points"[MeSH]) OR "Meridians"[MeSH]) |
| #2 | (((((((((( (acupuncture[Title/Abstract])) OR (acupuncture points[Title/Abstract])) OR (acupuncture therapy[Title/Abstract])) OR (body acupuncture[Title/Abstract])) OR (coiling dragon needling[Title/Abstract])) OR (dermal needle[Title/Abstract])) OR (dry needling[Title/Abstract])) OR (Electro-acupuncture[Title/Abstract])) OR (point injection[Title/Abstract])) OR (trigger points[Title/Abstract])) OR (meridians[Title/Abstract]) |
| #3 | (((((((((((((((chronic insomnia[MeSH Terms]) OR (familial fatal, insomnia[MeSH Terms])) OR (chronic insomnia[MeSH Terms])) OR (primary insomnia[MeSH Terms])) OR (Disorders of Initiating and Maintaining Sleep[MeSH Terms])) OR (insomnia[MeSH Terms])) OR (early awakening[MeSH Terms])) AND (awakening, early[MeSH Terms])) OR (nonorganic insomnia[MeSH Terms])) OR (insomnia, nonorganic[MeSH Terms])) OR (transient insomnia[MeSH Terms])) OR (rebound insomnia[MeSH Terms])) OR (dysfunction, sleep initiation[MeSH Terms])) OR (sleeplessness[MeSH Terms])) OR (insomnia disorder[MeSH Terms])) OR (insomnia, psychophysiological[MeSH Terms]) |
| #4 | "insomnia s"[All Fields] OR "sleep initiation and maintenance disorders"[MeSH Terms] OR ("sleep"[All Fields] AND "initiation"[All Fields] AND "maintenance"[All Fields] AND "disorders"[All Fields]) OR "sleep initiation and maintenance disorders"[All Fields] OR "insomnia"[All Fields] OR "insomnias"[All Fields] OR ("sleep wake disorders"[MeSH Terms] OR ("sleep"[All Fields] AND "wake"[All Fields] AND "disorders"[All Fields]) OR "sleep wake disorders"[All Fields] OR ("sleep"[All Fields] AND "disorder"[All Fields]) OR "sleep disorder"[All Fields]) |
| #5 | #1 OR #2 |
| #6 | #3 OR #4 |
| #7 | #5 AND #6 |

Search strategy for Web of Science:

Input "(TS=((acupuncture OR "acupuncture point*" OR "acupuncture therapy" OR "body acupuncture" OR "coiling dragon" OR "dermal needle" OR "dry needling" OR electroacupuncture OR "electro-acupuncture" OR "point injection" OR "trigger point*" OR meridian*))) AND (TS=((insomnia OR "chronic insomnia" OR "primary insomnia" OR "sleep initiation" OR "sleep maintenance" OR "early awakening" OR sleeplessness OR "sleep disorder*" OR "sleep initiation and maintenance disorders")))" into the "Advanced Search" section.

Search strategy for CNKI:

Input "SU=('针刺' + '针灸' + '穴位' + '经络' + '电针' + '干针' + '扳机点' + '腧穴' + '耳针' + '头针' + '温针' + '火针' + '针刀' + '穴位注射' + '腕踝针' + '腹针') AND SU=('失眠' + '不寐' + '睡眠障碍' + '失眠症' + '入睡困难' + '早醒' + '难寐' + '少寐')" into the Professional Search ("专业检索") section, and tick the synonym extension ("同义词扩展") choice.

Search strategy for VIP Database:

Input "(M=(针刺疗法 OR 穴位 OR 经络) OR T=(针刺 OR 针灸 OR 电针 OR 耳针 OR 头针 OR 干针 OR 针刀 OR 穴位注射 OR 腕踝针 OR 腹针 OR 触发点)) AND (M=(失眠 OR 睡眠障碍) OR T=(失眠 OR 不寐 OR 入睡困难 OR 早醒 OR 睡眠质量差))" into Professional Search ("专业检索"), and choose the tick of both bilingual extensions ("中英文扩展") and synonym extension ("同义词扩展").

Search strategy for Wanfang Database：

Input "((((K=针刺疗法 OR K=针刺 OR K=针灸 OR K=穴位 OR K=经络) OR (T=针灸 OR T=针刺 OR T=穴位 OR T=经络 OR T=电针 OR T=耳针 OR T=头针 OR T=干针 OR T=针刀 OR T=穴位注射 OR T=阿是穴 OR T=扳机点)) AND ((K=失眠 OR K=不寐 OR K=睡眠障碍 OR K=失眠症) OR (T=失眠 OR T=不寐 OR T=睡眠障碍 OR T=入睡困难 OR T=早醒))))" into Professional Search ("专业检索"), and choose the tick of both bilingual extensions ("中英文扩展") and subject term extensions ("主题词扩展").
