# Supplementary material for: Beyond nighttime symptoms: acupuncture for daytime dysfunction improvement in insomnia—a meta-analysis
Source: Front Neurol. 2026 Mar 3;17:1752313. doi: 10.3389/fneur.2026.1752313 (PMC12992216; doi:10.3389/fneur.2026.1752313)

PRISMA 2020 flow diagram for new systematic reviews which included searches of databases and registers only

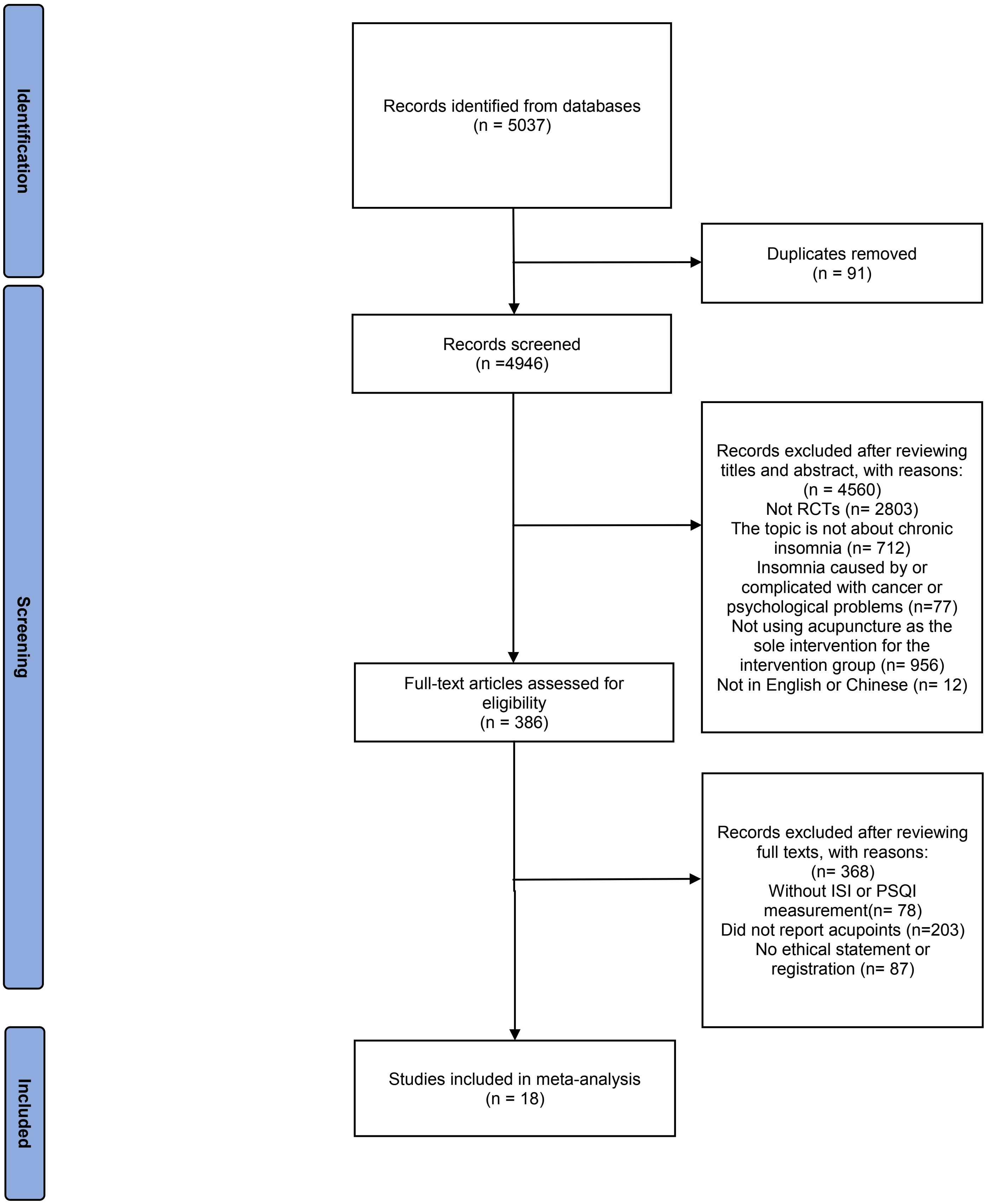

Supplement: Supplementary file 2 [file Data_Sheet_1.PDF]
