# Supplementary material for: Beyond nighttime symptoms: acupuncture for daytime dysfunction improvement in insomnia—a meta-analysis
Source: Front Neurol. 2026 Mar 3;17:1752313. doi: 10.3389/fneur.2026.1752313 (PMC12992216; doi:10.3389/fneur.2026.1752313)
Supplement: Supplementary file 3 [file Data_Sheet_2.PDF]

Study ID	D1	D2	D3	D4	D5	Overall
Chung KF et al., 2018	+	+	+	+	+	+
Pranab Das et al., 2022	+	+	+	!	+	!
Wu BX et al., 2023	+	+	+	+	+	+
Feng H et al., 2020	+	+	+	+	+	+
Wu WZ et al., 202109	+	+	+	!	+	!
Wu WZ et al., 202107	+	+	+	+	+	+
Cao JZ et al., 2023	+	+	+	!	+	!
Huo YS et al., 2023	+	+	+	+	+	+
Ding DM et al.,2023	+	+	+	+	+	+
Liu et al., 2024	+	+	+	+	+	+
Zhang et al., 2024	+	+	+	+	+	+
Guan et al., 2019	+	+	+	!	+	!
Wang et al., 2019	+	+	+	!	+	!
Yeung et al., 2009	+	+	+	+	+	+
Zhang et al., 2019	+	+	+	+	+	+
Wang XQ et al., 2021	+	+	+	+	+	+
Liu et al., 2025	+	+	+	+	+	+
Yu LJ et al., 2024	+	+	+	+	+	+

- + Low risk
- ! Some concerns
- - High risk

- D1 Randomisation process
- D2 Deviations from the intended interventions
- D3 Missing outcome data
- D4 Measurement of the outcome
- D5 Selection of the reported result
